# Supplementary material for: Facile orientation control of MOF-303 hollow fiber membranes by a dual-source seeding method
Source: Nat Commun. 2024 Nov 26;15:10264. doi: 10.1038/s41467-024-54730-z (PMC11599905; doi:10.1038/s41467-024-54730-z)
Supplement: Supplementary file 2 — Supplementary Information [file 41467_2024_54730_MOESM2_ESM.pdf]

Supplementary Information (SI)

Facile orientation control of MOF-303 hollow fibre membranes by a dual-source seeding method

Mengjiao Zhai[1,2], Farhad Moghadam[1,2], Tsaone Gosiamemang[2], Jerry Y.Y. Heng[2] and Kang Li[1,2]*

Barrer Centre, Chemical Engineering Department, Imperial College London, SW7 2AZ, UK

Department of Chemical Engineering, Imperial College London, London SW7 2AZ, UK

*Corresponding author E-mail: kang.li@imperial.ac.uk

Supplementary Methods

Materials: 1H-pyrazole-3,5-dicarboxylic acid (H_3PDC, ≥98%) and aluminum chloride hexahydrate ($AlCl_3 \cdot 6H_2O$, ≥99%) were purchased from Thermal Scientific. Sodium hydroxide (NaOH, ≥98%), methyl red (MR, MW 269.3), acetone, azobenzene (≥98%, 182.22), ethanol (absolute, >99.8%), 1-butanol (≥98.5%), N-methyl-2-pyrrolidone (NMP, anhydrous 99.5%), and poly(methyl methacrylate) (PMMA) were obtained from VWR. Protoporphyrin IX (PPh-IX, ≥99.5%, MW 562.66) and α-alumina powder (0.5-1 μm) were obtained from Inframat Advanced Materials. Arlacel P135 (polyethyleneglycol 30-dipolyhydroxystearate) was purchased from Uniqema. All chemicals were used as received without further purification.

Preparation of alumina support: alumina HFs were prepared based on a phase-inversion process by spinning a suspension containing Al_2O_3 (53.54 wt%), NMP (38.24 wt%), dispersant (0.57 wt%) and PMMA (7.65 wt%) followed by a sintering process at a temperature of up to 1450 °C[1]. The sintered HFs with the diameter of 2.23 mm were cut into 4 cm or 8 cm pieces and then washed with acetone and ethanol to remove impurities. The alumina disc used for measurements of contact angles was fabricated by casting the same suspension on a flat module, followed by the same sintering and washing process.

Synthesis of MOF-303 powder: MOF-303 powder was collected after membrane fabrication through the precipitation of one-step growth and secondary growth via the RS and DS methods via centrifugation. The collected powder was then washed with methanol three times and dried at 100 °C for further characterization.

Synthesis of the MOF-303 membrane by a liquid contact in situ growth method: MOF-303 synthesized by this method is illustrated in Supplementary Fig. 8. Briefly, 145 mg of H_3PDC and 67 mg of NaOH were dissolved in 27 mL of deionized water and sonicated for 15 min. The metal solution was prepared by mixing 100 mg of $AlCl_3 \cdot 6H_2O$ in 3 mL of deionized water in a separate container. The ligand and the metal solutions were simultaneously added to an autoclave with

HFs, which were sealed and placed vertically inside. Gentle stirring was required to obtain a homogenous solution. Then, the synthesis proceeded at 100 °C for 48 hours.

Synthesis of the MOF-303 membrane by a secondary growth method with a vacuum coating process for seeding: MOF-303 synthesized by this method is illustrated in Supplementary Fig. 9. MOF-303 seeds were dispersed in DI water via sonication at a concentration of 1 mg/mL. The dispersed MOF-303 suspension was coated on the outer surface of HFs using a vacuum filtration system. Two types of seeding layers with different thicknesses were formed by coating times of 10 s and 60 s, respectively. To improve the attachment between the coated seeding layer and the HF substrate, the mixture was kept under vacuum for 1 minute in air after the HFs were removed from the coating suspension and then dried in a vacuum oven at 40 °C for 4 hours. The secondary growth step of the vacuum-coated MOF-303 seeds was the same as that of the RS and DS processes.

Large-scale Synthesis of the MOF-303 membrane with DS method: The DS method was adapted for the large-scale synthesis of MOF-303 membranes with four 4 cm HF membranes in a single autoclave, and two 8 cm HF membranes in a larger autoclave with 2.5 times the volume (Supplementary Fig. 13).

Supplementary Figures

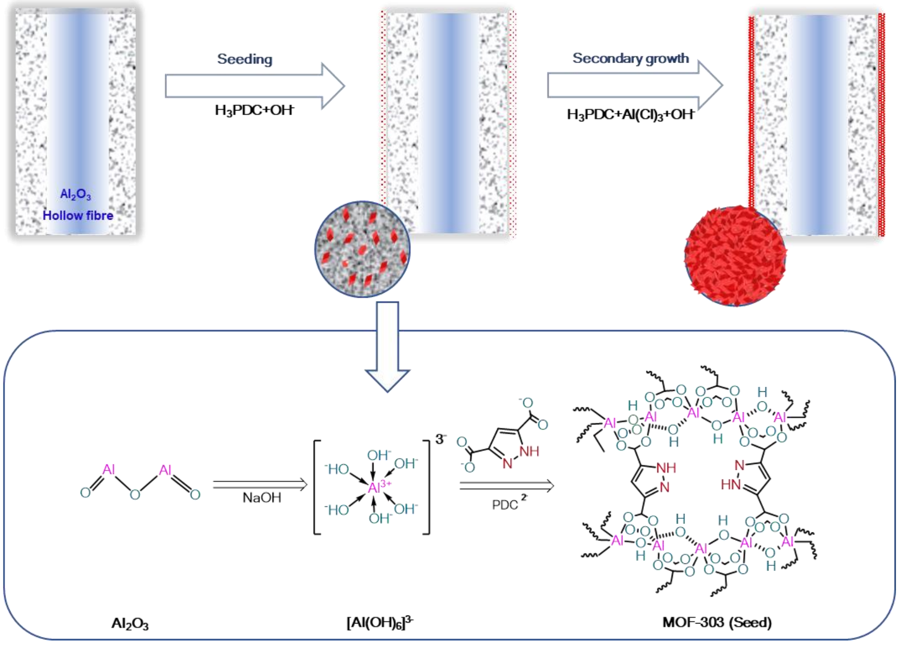

Supplementary Fig. 1 Schematic illustration of MOF-303 membranes synthesized by the conventional RS method. The alumina HF is placed vertically in an autoclave filled with ligand solution containing NaOH. The ligand reacts with the OH group on the surface alumina HF support to form the seed layer (seeding step), followed by a secondary growth for the final MOF-303 membranes named RS membrane.

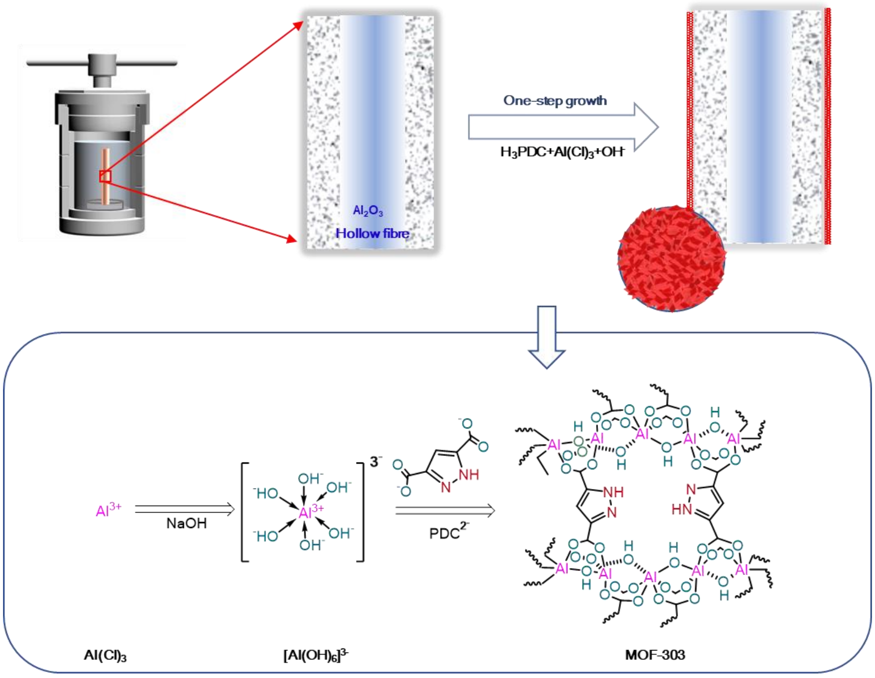

Supplementary Fig. 2 The schematic illustration of MOF-303 membranes synthesized by the conventional one-step method. In this method, the HF substrate is vertically put into the reaction solution including ligand, metal salt and NaOH to form the resultant MOF membrane. The metal source with sufficient stochiometric ratio in the reaction solution is readily available to react with ligand and instantly form crystals.

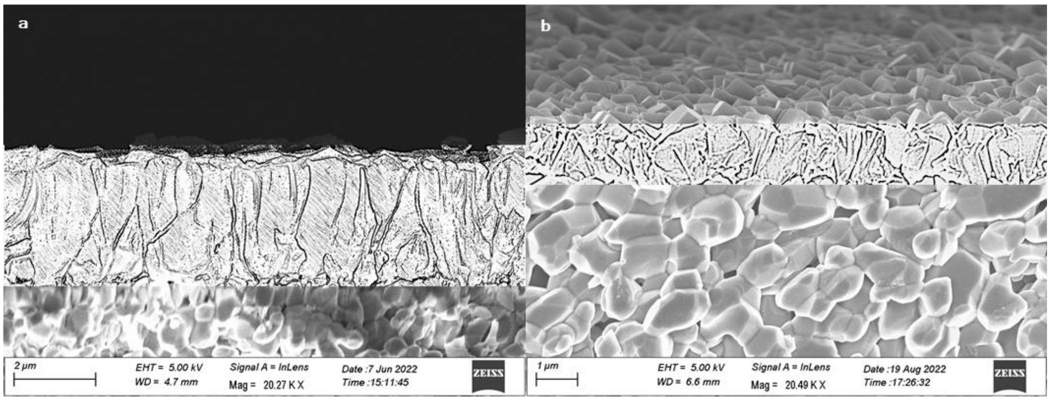

Supplementary Fig. 3 Sketchy view of the cross-section of membrane fabricated by DS method after **a,** 48 hours growth, and **b**, 1 hour growth, obtained by processing the original SEM image in Photoshop to highlight the outline of the crystals.

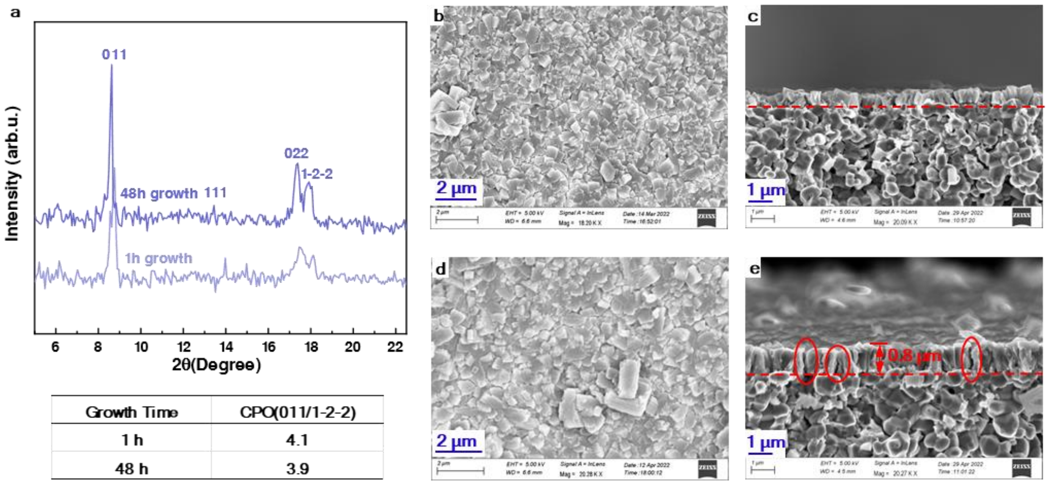

Growth Time	CPO(011/1-2-2)
1 h	4.1
48 h	3.9

Supplementary Fig. 4 SEM images and XRD patterns of MOF-303 membranes fabricated by conventional one-step method. a XRD patterns and corresponding CPO ratio of membrane. The patterns match those of MOF-303 powder. The SEM images of the membrane after **b, c** 1-hour synthesis and **d, e** 48-hour synthesis. The red circles in **e** display the intercrystalline gaps and interfacial voids between the substrate and MOF-303 layer. Red lines are to distinguish the MOF-303 layer from the alumina substrate.

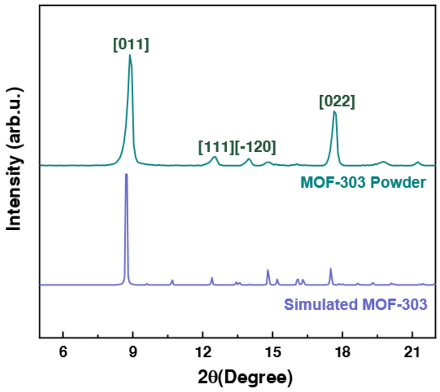

Supplementary Fig. 5 XRD patterns of simulated MOF-303 and the MOF-303 powder collected during the growth.

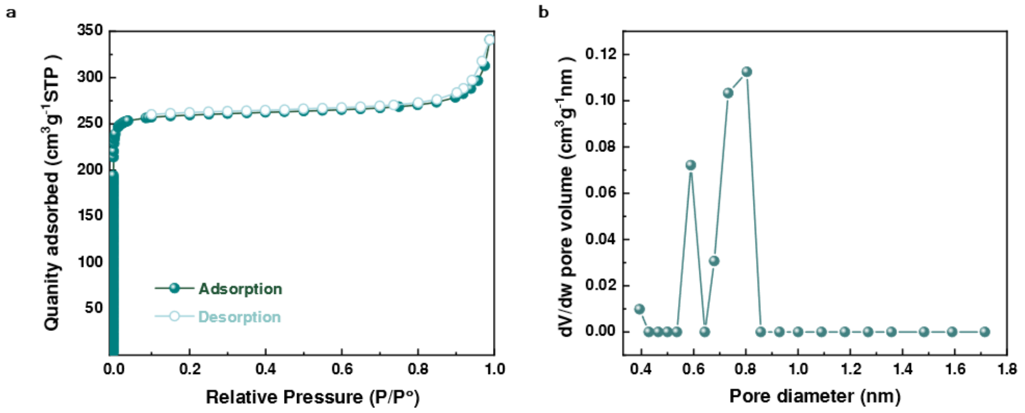

Supplementary Fig. 6 BET analysis of MOF-303. a N_2 sorption isotherms of MOF-303 powder and **b** corresponding pore size distribution. The MOF-303 crystals contain pores with the size of 0.6-0.8 nm.

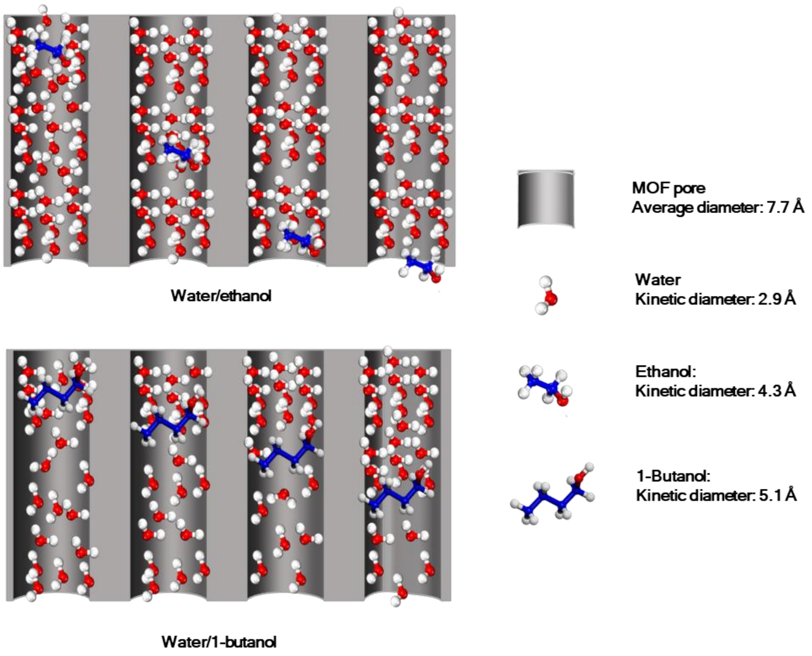

Supplementary Fig. 7 Schematic illustration of the transport of water/ethanol and water/1-butanol mixture in the MOF-303 membrane.

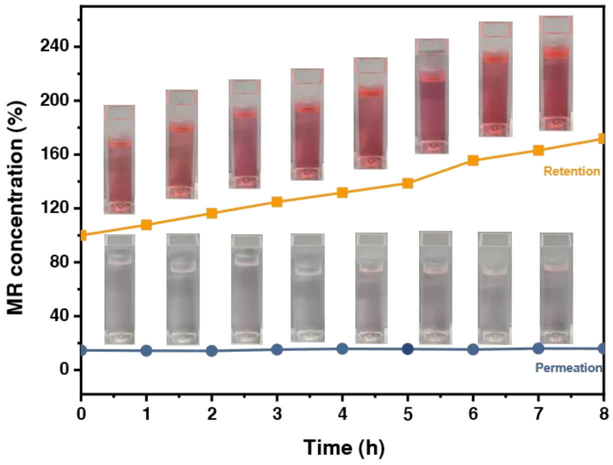

Supplementary Fig. 8 Concentration changes during long-term MR rejection tests of DS membranes.

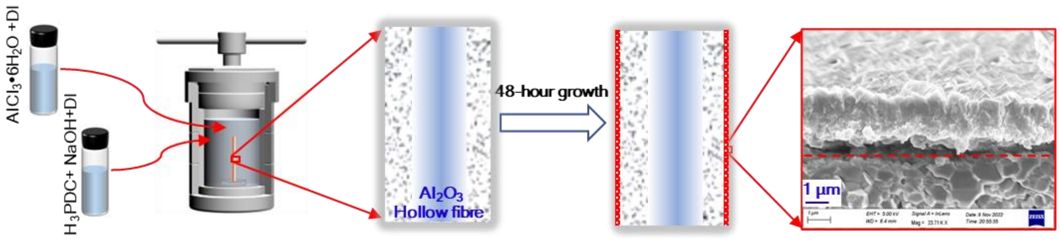

Supplementary Fig. 9 The schematic illustration of MOF-303 membranes synthesized by the liquid contact method. In the liquid contact method, the ligand with NaOH and metal salt dissolved separately before adding to the autoclave. The instant reaction formed a membrane with an amorphous structure in the bottom part and poor attachment with the substrate.

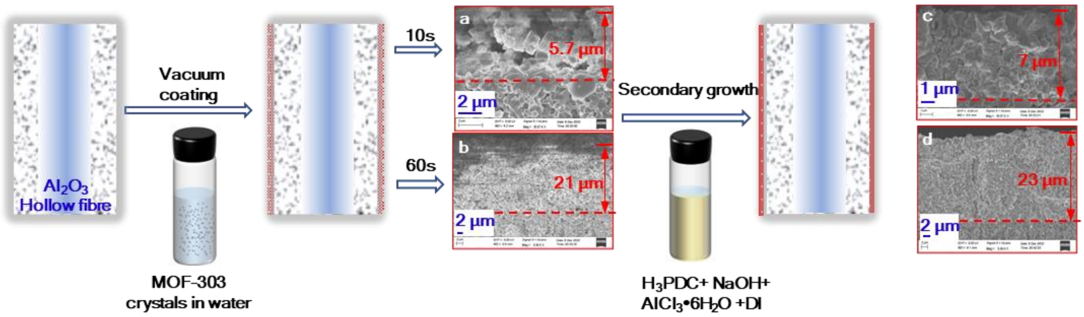

Supplementary Fig. 10 The schematic illustration of MOF-303 membranes synthesized by the secondary growth of vacuum-coated seeding layer. The MOF-303 crystals was dispersed in water and vacuum-coated onto HF substrate to form the seeding layer. The thickness of the seeding layer was adjusted by varying the coating time. This formed **a** a 5.7 μm seeding layer by10 seconds coating, and **b** a 21 μm seeding layer by 60 seconds coating. These seeding layers subsequently grew into final membranes with thicknesses of **c** 7 μm, and **d** 23 μm, respectively.

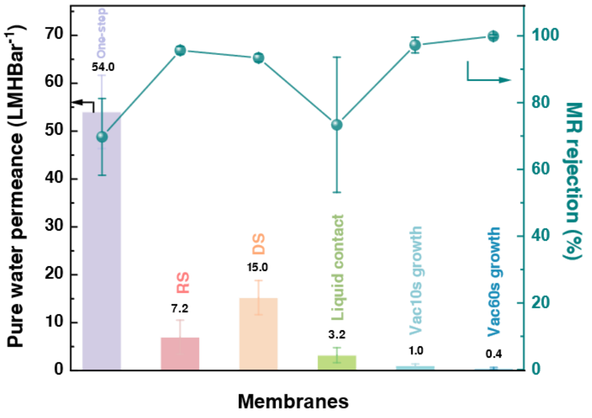

Supplementary Fig. 11 Pure water permeance of MOF-303 membranes prepared by different methods. Comparison of pure water permeance and MR (269.3 g mol^{-1}) rejection of one-step, DS, and RS MOF-303 membranes with those fabricated by liquid contact (tested at 5 bar) and vacuum-coated seeded growth techniques (tested at 10 bar). Error bars represent the standard deviation (n=3).

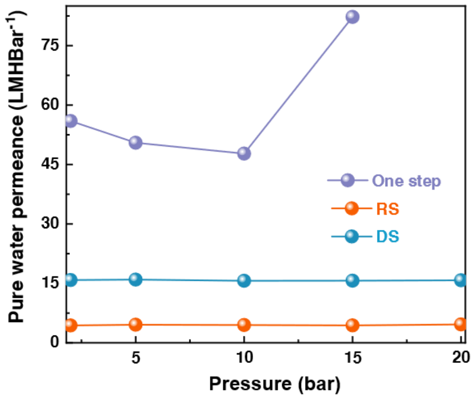

Supplementary Fig. 12 Pure water permeance of MOF-303 membranes measured at different pressures by dead-end filtration system.

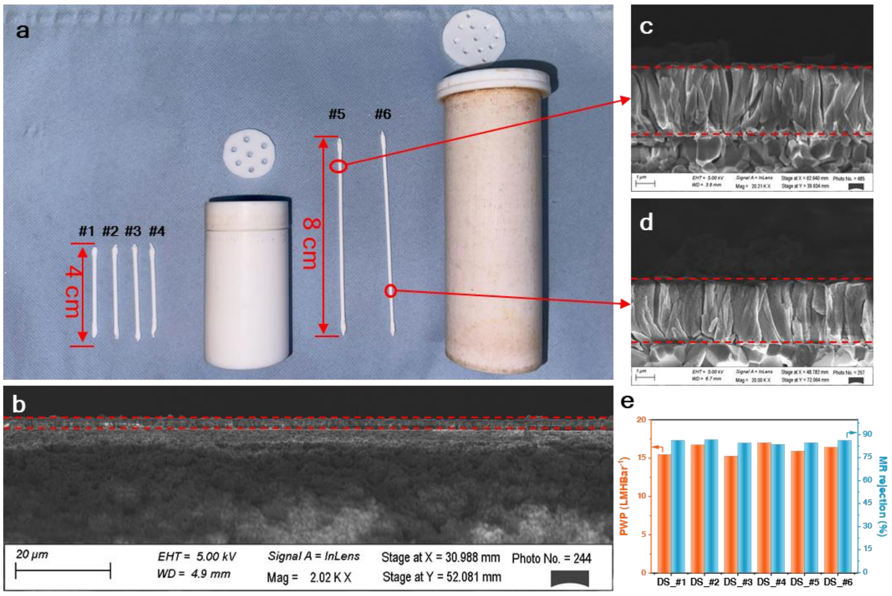

Supplementary Fig. 13 Samples fabricated at a larger scale. **a** HFs were cut into lengths of 4 cm and 8 cm for the fabrication of DS membranes in autoclaves of different sizes. **b, c** and **d** Cross-sectional views of the DS membrane observed at random positions in samples fabricated in the larger autoclave. **e** PWP and MR rejection of five samples fabricated in different autoclaves, Among them, DS_#1-4 are 4 cm samples synthesized in a single autoclave, while DS_#5 and DS_#6 are 8 cm samples synthesized in a larger volume autoclave. Red lines are to distinguish the MOF-303 layer from the alumina substrate.

.

Supplementary Tables

Supplementary Table 1 Mass flux and water/EtOH separation factor of pristine MOF and other state-of-the-art membranes for pervaporation.

**Material**	**Feed solution**	**Temperature (K)**	**Flux ($kg\ m^{-2}\ h^{-1}$)**	**Separation factor (-)**	**Reference number in the main text**
MOF-303(RS)	95 wt% EtOH	343	6.1 ± 0.47	188 ± 3.52	**This work**
MOF-303(DS)	95 wt% EtOH	343	17.9 ± 2.85	44 ± 1.50	**This work**
CAU-10-H	90 wt% EtOH	338	0.6	148	2
Sm-DOBDC	92 wt% EtOH	298	0.6	997	3
UiO-66	90 wt% EtOH	343	3.7	55	4
UiO-66	10 wt% EtOH	323	1.5	4.9	5
$Ni_2(L\text{-}asp)_2bipy$	90 wt% EtOH	333	0.6	0.1	6
MOF-303 PDC-TDC	90 wt% EtOH		1	8,500	7
MOF-303-Urea	90 wt% EtOH	343	0.2	1,874	8
ZIF-71	5 wt% EtOH	323	6.1	5.0	9
ZIF-71	5 wt% EtOH	298	0.3	6.1	10
SIM-1	50 wt% EtOH	298	0.5	∞	11
NU-906	90 wt% EtOH	353	0.1	244	12
LTA	90 wt% EtOH	343	1.2	18,000	13
LTA	90 wt% EtOH	348	3.7	>10,000	14
HLTA	90 wt% EtOH	348	4.5	>10,000	14
ERI	90 wt% EtOH	343	0.9	1,000	13
MFI	95 wt% EtOH	298	2.9	160	15
MFI	95 wt% EtOH	333	9.8	58	16
MFI	95 wt% EtOH	333	1.9	64	17

ZSM-5	90 wt% EtOH	323	0.7	200	[18]
SSZ-13	90 wt% EtOH	333	1.3	>10,000	[19]
NaA	90 wt/% EtOH	343	2.8	>10,000	[20]
NaA	90 wt/% EtOH	348	8.5	>10,000	[21]
NaA	90 wt/% EtOH	348	12.8	10,000	[22]
NaA	90 wt/% EtOH	348	3.8	73,800	[23]
NaA	90 wt% EtOH	348	11.1	>10,000	[24]
CHA	90 wt/% EtOH	348	14	>10,000	[25]
CHA	90 wt% EtOH	348	1.2	>10,000	[26]
CHA	90 wt% EtOH	348	1.2	5,400	[27]
CHA	90 wt% EtOH	355	14.5	15,000	[25]
FAU	92 wt% EtOH	338	1.7	10,000	[28]
NaX	90 wt% EtOH	338	3.4	296	[29]
MOR	90 wt% EtOH	298	9.1	990	[30]
GIS	90 wt% EtOH	348	0.3	200,000	[31]
Zeolite T	90 wt% EtOH	348	1.2	>10,000	[32]
Zeolite T	90 wt% EtOH	348	1.3	2,100	[33]
Zeolite T	90 wt% EtOH	348	1.4	3,100	[34]
Zeolite T	90 wt% EtOH	348	2.3	1,348	[35]
GO	90 wt% EtOH	363	0.3	299	[36]
Graphene oxide framework	90 wt% EtOH	323	0.6	10,000	[37]
Graphene oxide framework	85 wt% EtOH	333	0.8	56.6	[38]
GO/cellulose acetate	90 wt% EtOH	353	2.3	2,241	[39]
rGO/Sodium alginate	90 wt% EtOH	349	1.4	1,500	[40]
rGO/polyvinyl alcohol	90 wt%EtOH	323	0.4	12,000	[41]

GO/polyvinyl alcohol	90 wt% EtOH	323	0.3	115.5	42
GO/polyvinyl alcohol	90 wt% EtOH	313	0.1	263	43
GO-carbon nanotubr/polyvinyl alchol	90 wt% EtOH	296	0.9	523	44
GO/polyethyleneimine	98 wt% EtOH	333	1.8	77	45
GO/Sodium alginate/polyethyleneimine	90 wt% EtOH	295	0.2	8,991	46
Carbon nanotube/polyvinyl alcohol	70 wt% EtOH	298	0.9	4,464	47
UiO-66/GO	98 wt% EtOH	323	3.2	6,951	48
ZIF-8@GO /polydimethylsiloxane	5 wt% EtOH	313	0.4	22.2	49
ZIF-8/sodium alginate	90 wt% EtOH	349	2.5	1,884	50
ZIF-8/sodium alginate	90 wt% EtOH	349	0.9	678	51
ZIF-L/sodium alginate	90 wt% EtOH	349	1.2	1,840	51
Zn/Co-ZIF/polymer of intrinsic microporosity-1	5 wt% EtOH	338	1.2	6.4	52
CAU-10/chitosan	90 wt% EtOH	298	0.6	1,369	53
MAF-6/poly (ether-block-amide)	5 wt% EtOH	333	4.4	5.6	54
UiO-66-NH_2/polyimide	85 wt% EtOH	333	0.7	142	55
Co-MOF/sodium alginate	90 wt% EtOH	-	1.9	800	56
HKUST-1/polyimide	90 wt% EtOH	315	0.2	200	57
MOF-801/polyvinyl alcohol	90 wt% EtOH	343	3.6	283	58
MO-801/chitosan	90 wt% EtOH	323	1.9	2,156	59
NU-906/chitosan	90 wt% EtOH	349	1.1	2,651	60
LZU-8/polydimethylsiloxane	95 wt% EtOH	-	5	11	61

SUZ-4/polyamide	90 wt%EtOH	333	3.2	1,056	[62]
ZSM-5/polyvinyl alcohol	80 wt% EtOH	323	1	660	[63]
Zeolite/polyvinyl alcohol	80 wt% EtOH	333	1.7	360	[64]
COF TpHZ/poly(ether sulfone)	90 wt% EtOH	349	2.5	1,430	[65]
COF TpEB/sodium alginate	90 wt% EtOH	349	2.5	2,110	[66]
TpBD COF	90 wt% EtOH	349	2.2	2,099	[67]
$Ti_3C_2T_x$MXene	95 wt% EtOH	343	0.3	135	[68]
MXene/cellulose acetate	90 wt% EtOH	313	1.4	1,421	[69]
MXene/chitosan	98 wt% EtOH	323	1.2	906	[70]
MXene/polyvinyl alcohol	95 wt% EtOH	303	1.2	1,577	[71]
MXene/sodium alginate	90 wt% EtOH	343	1.4	1,650	[72]
MXene/sodium	90 wt% EtOH	343	0.5	9,946	[73]
MoS_2/sodium alligate	50 wt% EtOH	349	1.8	1,229	[74]
C_3N_4/poly(vinyl alcohol)	90 wt% EtOH	348	6.7	30.7	[75]
Carbon nanotubes/poly(vinyl alcohol) /polyethersulfone	90 wt% EtOH	303	0.5	805	[76]
Polyvinyl alcohol/sodium alginate/polyacrylonitrile	90 wt% EtOH	342	1.2	5,164	[77]
Sodium alginate/polyvinylidene fluoride	90 wt% EtOH	298	1	2,638	[78]
Sodium alginate/polyamide	85 wt% EtOH	343	2	525.1	[79]
Sodium alginate/polyamide	85 wt% EtOH	333	1.5	297	[80]
Chitosan/Fe^{3+}-phytic acid	85 wt% EtOH	328	2.9	1,128	[81]
Polydimethylsiloxane	95 wt% EtOH	323	1.1	8.2	[82]
Siloxane/chitosan	90 wt% EtOH	298	0.5	2,182	[83]
Poly(allylamine hydrochloride) /polyvinyl alcohol /trimesic acid	90 wt% EtOH	343	1.3	5,285	[84]

Poly(4-styrenesulfonic acid)/chitosan	90 wt% EtOH	343	0.5	904	[85]
Polyamide	90 wt% EtOH	349	4.4	3870	[86]
Sodium alginate	75 wt% EtOH	348	1.3	187	[87]
Carbon molecular sieve	90 wt/% EtOH	343	0.5	1,946	[88]

Supplementary Table 2 Mass flux and water/1-butanol separation factor of pristine MOF and other state-of-the-art membranes for pervaporation.

Material	Feed solution	Temperature (K)	Flux (kg m^{-2} h^{-1})	Separation factor (-)	Reference number in the main text
MOF-303(RS)	95 wt% 1-BuOH	343	2.8 ± 0.76	10,142	**This work**
MOF-303(DS)	95 wt% 1-BuOH	343	7.3 ± 1.64	4,636	**This work**
UiO-66	90 wt% 1-BuOH	303	5.4	4,280	[4]
fum-Zr-MOF	95 wt% 1-BuOH	313	3.6	1,653	[89]
NU-906	90 wt% 1-BuOH	353	1.6	2,630	[12]
Ni-MOF-74	90 wt% 1-BuOH	333	1.8	1,094	[90]
Beta	90 wt% 1-BuOH	348	2.8	17,900	[91]
Beta	90 wt% 1-BuOH	348	3	22,000	[92]
Beta	1 wt% 1-BuOH	318	1	1.3	[93]
NaY	90 wt% 1-BuOH	348	2.6	1,000	[94]
NaY	95 wt% 1-BuOH	348	2	1,500	[94]
Fe-beta	95 wt% 1-BuOH	303	2.1	6.2	[95]
NaA	96 wt% 1-BuOH	393	1.5	200	[96]
ZIF-7/polydimethylsiloxane	1 wt% 1-BuOH	333	1.6	44	[97]
ZIF-71/ polydimethylsiloxane	2 wt% 1-BuOH	333	-	69.9	[98]
ZIF-90/sodium alligate	60 wt% BuOH	313	1.6	1,678	[99]
Zn/Co-ZIF/polymer of intrinsic microporosity-1	95 wt% BuOH	338	1.7	21.3	[52]

UiO-66/polyvinyl alcohol	90 wt% 1-BuOH	343	1.2	2,110	[100]
UiO-66/polyimide	85 wt% 1-BuOH	333	0.2	12,214	[101]
Silicon/1, 2-bis(triethoxysilyl) ethane	90 wt% 1-BuOH	323	1.1	19	[102]
Zeolite A/polyvinyl alcohol/chitosan	90 wt% 1-BuOH	303	13.7	1,324	[103]
ZIF-8/GO	90 wt% BuOH	343	5.3	3,567	[104]
COF TpHZ	90 wt% 1-BuOH	353	8.2	1,023	[105]
COF TpHZ@CTpDHBD	90 wt% 1-BuOH	353	14.4	4,464	[106]
COF TaPa@Hz	90 wt% 1-BuOH	353	11.4	3,620	[107]
COF T_aP_a-1/sodium alginate	90 wt% 1-BuOH	313	1.8	4,687	[108]
COF $TpTG_{c1}$/ Cellulose nanofibers	90 wt% 1-BuOH	353	8.5	3,876	[109]
COF LZU1/ poly(ether-block-amide)	3.7 wt% 1-BuOH	307	0.6	22.2	[110]
Calcium alginate/COF TpHZ	90 wt% 1-BuOH	349	3.6	2,764	[111]
Graphene oxide framework	85 wt% 1-BuOH	333	2.6	1,883	[38]
GO	90 wt% 1-BuOH	333	4.8	5,705	[112]
GO	90 wt% 1-BuOH	353	9.1	2,941	[113]
Sulfobutyl-beta-cyclodextrin/GO	90 wt% 1-BuOH	353	13	1,299	[114]
Vermiculite/GO	10 wt% 1-BuOH	353	9.6	2,678	[115]
Carbon quantum dots/GO	90 wt% BuOH	343	5.9	4,470	[116]
Carbon quantum/GO	90 wt% BuOH	298	1.6	1,376	[117]

Imidazole-ureido/GO	80 wt% 1-BuOH	343	3.5	4,454	[118]
SiO_2/polyimide	85 wt% 1-BuOH	313	0.1	279	[119]
SiO_2/chitosan	90 wt% BuOH	323	0.7	1,498	[120]
Poly(1-trimethylsilyl-1-propyne)/silica	1.5 wt% 1-BuOH	336	0.2	126	[121]

Supplementary Table 3 Comparison of nanofiltration performance of MOFs-based membranes

MOF	Polymer matrix	Fabrication technique	Permeance (L m^{-2} h^{-1} bar^{-1})	Rejection	Ref.
**MOF -303**	**-**	**RS**	**7.0**	**Azobenzene, 80.2%**<br>**Methyl red, 95.7%**<br>**Disperse red, 100%**<br>**Protoporphyrin IX, 100%**	**This work**
**MOF -303**	**-**	**DS**	**15.2**	**Azobenzene, 61.2%**<br>**Methyl red, 93.4%**<br>**Disperse red, 97.2%**<br>**Protoporphyrin IX, 100%**	**This work**
MOF -303	-	In-situ growth	0.7	$MgCl_2$, 93.5%<br>Na_2SO_4, 96.0%	122
ZIF-8	-	Nanoreactor-confined crystallization	130	Proanthocyanidin, 98.5%	123
ZIF-8	-	In-situ growth on a tannic acid (TA) and Zn^{2+} network	5.1	NaCl, 55.2%<br>Na_2SO_4, 93.6%	124
ZIF-8	Polyamide	Liquid-liquid interfacial coordination	37.5	Rose Bengal, >98%	125
ZIF-8	Polyamide	In situ growth with substrate modification	>30	Methyl orange, >97%<br>Isatin, >97%<br>Methyl blue, >99%<br>Congo red, >99%	126
ZIF-8	Polyamide	Layer-by-layer	27	Congo red, >99.8%	127
ZIF-8	Polyamide	Non-solvent induced phase inversion	10.4	Rode Bengal, >96.1%	128
ZIF-8	Polyamide	Interfacial polymerization	4.8	SO_4^{2-}, 89.9%	129
ZIF-8	Polyamide	Layer-by-layer	5.5	Acetaminophen, 55%	130
ZIF-8	Polyethyleni mine	Self-assembly and interfacial reaction	33	Methyl blue, 99.6%	131
ZIF-8	Polyamide	Blending interfacial polymerization	7.1	NaCl, 35%<br>Xylose, 67%	132
ZIF-300	-	Secondary growth	39.2	Rhodamine B, 99.9%<br>Methyl blue, 99.6%<br>Methyl orange, 98.89%	133

ZIF-L	-	Heteroepitaxial growth	51.6	Methyl orange, 80%<br>Acid fuchsin, 90%	134
UiO-66	-	In-situ growth	0.2	Ca^{2+}, 86.3%<br>Mg^{2+}, 98. 0%<br>Al^{3+}, 99.3%	135
UiO-66-OH_2	-	Secondary growth	2.4	Methyl blue, 98.7%<br>Na^{+}, 26%<br>Zn^{2+}, 42.5%<br>Fe^{3+}, 54.7%	136
NH_2-UiO-66@ZIF-8	-	In-situ growth	36.7	Direct red 80, 100%<br>Acid orange, 100%<br>Methyl orange, 99.6%<br>Methylene blue, 97.0%	137
UiO-66	GO/polyacrylonitrile	Vacuum filtration after phase inversion	31.3	Methyl orange, 94.8%<br>Methyl blue, 100%<br>Congo red, 99.6<br>Rhodamine B, 95.5<br>Tetracycline hydrochloride, 95.5%<br>Oxytetracycline, 94.8%<br>Ciprofloxacin, 98.6%	138
UiO-66-NH_2	Polyamide	Blending interfacial polymerization	7.2	NaCl, 42%<br>Xylose, 65%	132
UiO-66	Polyamide	Dispersion and interfacial polymerization	11.5	SeO_3^{2-}, 96.5%<br>SeO_4^{2-}, 97.4%<br>$HAsO_4^{2-}$, 98.6%	139
UiO-66-NH_2	Polyamide	Blending and interfacial polymerization	30	Methyl orange, 92%<br>Sunset yellow, 96%<br>Congo red, 99.6%	140
UiO-66	Polyelectrolytes	Blending and layer-by-layer	14.8	$MgSO_4$, 96.3%<br>Congo red, 99.9%	141
UiO-66-NH_2	Polyacrylonitrile	Drop coating and vacuum filtration	14	Congo red, 94%<br>Methyl orange, 94%<br>Rhodamine B, 94%<br>Methylene blue, 94 %	142
UiO-66-NH_2	Graphene oxide	Crosslinking and vacuum filtration	83.5	Methyl blue, 100%<br>Congo red, 99.9%<br>Alphazurine A, 97.8%<br>Eriochrome Black T, 99.3<br>Crystal violet, 100<br>Disperse black 9, 100%	143
MIL-53(Al)	Polyamide	Blending interfacial polymerization	6.9	NaCl, 40%<br>Xylose, 61%	132
NH_2-MIL-101(Al)	Chitosan	Blending and solvent evaporation	4	$MgCl_2$, 93.0%<br>$CaCl_2$, 86.5%	144
BUT-8(A)	Polyethylenimine	Blending and spinning coating	Up to 68.3	Methyl blue, 98.3%<br>Congo red, 99.8%<br>Acid fuchsin, 89.3%	145

				Methyl orange, 82.1%	
BUT-8(A)	Polydiallyldimethylammonium chloride	Spinning coating	16.4	Methyl blue, 98.8%	146
$Zn_2(bim)_4$	Polyethylenimine	Blending and vacuum coating	290	Methyl red, 98%<br>Coomassie brilliant blue, 98%<br>Methylene blue, 98%<br>Ca^{2+}, 66.2%<br>Mg^{2+}, 52.7%<br>Al^{3+}, 74.3%	147
MOF-1	Chitosan	Blending and crosslinking	20	Metyl blue, 85%<br>Rhodamine B, 85%<br>Eriochrome black t, 92%<br>CR: 98	148
MOF-801	-	Epitaxial growth	>71	Congo red, 99.8%<br>Methyl blue, 99.7%<br>Malachite green, 99.4%	149
MOF-808	-	In-situ growth	3.6	Congo red, 99.5%	150
MIP-202	GO	Blending and vacuum filtration	55.3	Crystal violet, 99.5%	151
NUS-8	-	Doctor-blading	~1	Mg^{2+}, 98%<br>Al^{3+}, 98%<br>Acide fuchsin, ~ 90%<br>Methyl blue, ~94%	152
CuBTC	Polyvinyl alcohol	In-situ growth and crosslinking	12.9	Congo red, 99.5%	153
Cu-TCPP	-	Vacuum filtration	840.1	Congo red, 99.8%<br>Chrome black T, 93.3%<br>Methyl blue, 99.7%<br>Crystal violet, 90.1%	154
Cu-TCPP	-	Electrophoretic deposition	16.4	Brilliant blue G, 97%	155
Cu-TCPP	-	Vacuum filtration	9.4	Evans blue, 97%	155
Ag-MOF	Polyamide	Vacuum assisted interfacial polymerization	14.3	Methyl orange, 95.4%<br>Methyl blue, 88.7%<br>Rhodamine B, 99.3%<br>Na_2SO_4, 84.1%<br>$MgSO_4$, 73.1%	156
Ni-MOF	-	Vacuum filtration	-	Congo red, 97.7%	157
Prussian blue	-	In-situ growth	26.2	Congo red, 99.7%<br>Calcein, 99.3%<br>Methyl blue, 99%	158

Supplemental References

1. Wu T, Prasetya N, Li K. Re-generable and re-synthesisable micro-structured MIL-53 Rachig Rings for ibuprofen removal. *J. Environ. Chem. Eng.* **10**, 107432 (2022).

2. Jin H, Mo K, Wen F, Li YJJoms. Preparation and pervaporation performance of CAU-10-H MOF membranes. *J. Membr. Sci.* **577**, 129-136 (2019).

3. Zhai L*, et al.* Polycrystalline rare-earth metal-organic framework membranes with in-situ healing ability for efficient alcohol dehydration. *J. Membr. Sci.* **610**, 118239 (2020).

4. Liu X, Wang C, Wang B, Li K. Novel Organic-Dehydration Membranes Prepared from Zirconium Metal-Organic Frameworks. *Adv. Funct. Mater.* **27**, 1604311 (2017).

5. Miyamoto M, Hori K, Goshima T, Takaya N, Oumi Y, Uemiya S. An Organoselective Zirconium-Based Metal–Organic-Framework UiO-66 Membrane for Pervaporation. *Eur. J. Inorg. Chem.* **2017**, 2094-2099 (2017).

6. Wang S*, et al.* Wettability switchable metal-organic framework membranes for pervaporation of water/ethanol mixtures. *Inorganic Chem. Comm.* **82**, 64-67 (2017).

7. Hu F-H*, et al.* Mixed-linker MOF-303 membranes for pervaporation. *J. Membr. Sci. Lett.* **3**, 100053 (2023).

8. Lai J-Y, Wang T-Y, Zou C, Chen J-J, Lin L-C, Kang D-Y. Highly-selective MOF-303 membrane for alcohol dehydration. *J. Membr. Sci.* **661**, 120879 (2022).

9. Huang K, Li Q, Liu G, Shen J, Guan K, Jin W. A ZIF-71 Hollow Fiber Membrane Fabricated by Contra-Diffusion. *ACS Appl. Mater. Interfaces* **7**, 16157-16160 (2015).

10. Dong X, Lin YS. Synthesis of an organophilic ZIF-71 membrane for pervaporation solvent separation. *Chem. Comm.* **49**, 1196-1198 (2013).

11. Marti AM, Tran D, Balkus KJJJoPM. Fabrication of a Substituted Imidazolate Material 1 (SIM-1) membrane using post synthetic modification (PSM) for pervaporation of water/ethanol mixtures. *J. Porous Mater.* **22**, 1275-1284 (2015).

12. Luo R*, et al.* In Situ Fabrication of Metal–Organic Framework Thin Films with Enhanced Pervaporation Performance. *Adv. Funct. Mater.* **33**, 2213221 (2023).

13. Sommer S, Melin T. Performance evaluation of microporous inorganic membranes in the dehydration of industrial solvents. Chem. Eng. Process **44**, 1138-1156 (2005).

14. Zhang H, Yang Y, Wang Z. Synthesis of hierarchical LTA zeolite membranes by vapor phase transformation. *J. Membr. Sci.* **671**, 121391 (2023).

15. Mirfendereski SM, Lin JYS. High-performance MFI zeolite hollow fiber membranes synthesized by double-layer seeding with variable temperature secondary growth. *J. Membr. Sci.* **618**, 118573 (2021).

16. Xia S, Peng Y, Wang Z. Microstructure manipulation of MFI-type zeolite membranes on hollow fibers for ethanol–water separation. *J. Membr. Sci.* **498**, 324-335 (2016).

17. Peng Y, Zhan Z, Shan L, Li X, Wang Z, Yan Y. Preparation of zeolite MFI membranes on defective macroporous alumina supports by a novel wetting–rubbing seeding method: Role of wetting agent. *J. Membr. Sci.* **444**, 60-69 (2013).

18. Wang Q*, et al.* Synthesis optimization and separation mechanism of ZSM-5 zeolite membranes for pervaporation dehydration of organic solvents. *Sci Total Env.* **929**, 172641 (2024).

19. Yi R*, et al.* Effect of the Si/Al Ratio of the Seeds in the Preparation of Supported SSZ-13 Membranes with a Secondary Growth Method. *Ind. Eng. Chem. Res.* **62**, 17893-17904 (2023).

20. Kunnakorn D*, et al.* Optimization of synthesis time for high performance of NaA zeolite membranes synthesized via autoclave for water–ethanol separation. *Desalination* **280**, 259-265 (2011).

21. Charik FZ*, et al.* Optimal preparation of low-cost and high-permeation NaA zeolite membrane for effective ethanol dehydration. *Microporous Mesoporous Mater.* **344**, 112229 (2022).

22. Liu D, Zhang Y, Jiang J, Wang X, Zhang C, Gu X. High-performance NaA zeolite membranes supported on four-channel ceramic hollow fibers for ethanol dehydration. *RSC Adv.* **5**, 95866-95871 (2015).

23. Hasegawa Y, Matsuura W, Abe C, Ikeda A. Influence of Organic Solvent Species on Dehydration Behaviors of NaA-Type Zeolite Membrane. *Membranes* **11**, 347 (2021).

24. Shao J, Zhan Z, Li J, Wang Z, Li K, Yan Y. Zeolite NaA membranes supported on alumina hollow fibers: Effect of support resistances on pervaporation performance. *J. Membr. Sci.* **451**, 10-17 (2014).

25. Hasegawa Y, Abe C, Nishioka M, Sato K, Nagase T, Hanaoka T.

Formation of high flux CHA-type zeolite membranes and their application to the dehydration of alcohol solutions. *J. Membr. Sci.* **364**, 318-324 (2010).

26. Du J*, et al.* Template-Free Synthesis of High Dehydration Performance CHA Zeolite Membranes with Increased Si/Al Ratio Using SSZ-13 Seeds. *Membranes* **14**, 78 (2024).

27. Hasegawa Y, Abe C, Ikeda A. Pervaporative Dehydration of Organic Solvents Using High-Silica CHA-Type Zeolite Membrane. *Membranes* **11**, 229 (2021).

28. Zhu G, Li Y, Zhou H, Liu J, Yang W. Microwave synthesis of high performance FAU-type zeolite membranes: Optimization, characterization and pervaporation dehydration of alcohols. *J. Membr. Sci.* **337**, 47-54 (2009).

29. Zhou H, Korelskiy D, Leppäjärvi T, Grahn M, Tanskanen J, Hedlund J. Ultrathin zeolite X membranes for pervaporation dehydration of ethanol. *J. Membr. Sci.* **399-400**, 106-111 (2012).

30. Alomair AA, Alqaheem Y. Optimization of Mordenite Membranes Using Sucrose Precursor for Pervaporation of Water-Ethanol Mixtures. *Membranes* **11**, 160 (2021).

31. Guo J-C*, et al.* NaP1 zeolite membranes with high selectivity for water-alcohol pervaporation. *J. Membr. Sci.* **639**, 119762 (2021).

32. Zhou H, Li Y, Zhu G, Liu J, Yang W. Preparation of zeolite T membranes by microwave-assisted in situ nucleation and secondary growth. *Mat. Lett.* **63**, 255-257 (2009).

33. Kondo M, Kita H. Permeation mechanism through zeolite NaA and T-type membranes for practical dehydration of organic solvents. *J. Membr. Sci.* **361**, 223-231 (2010).

34. Shafiei K, Pakdehi SG, Moghaddam MK, Mohammadi T. Improvement of Zeolite T Membrane via Clear Solution Gel in Dehydration of Methanol, Ethanol, and 2-Propanol. *Sep. Sci.Technol.* **49**, 797-802 (2014).

35. Ji M, Gao X, Wang X, Zhang Y, Jiang J, Gu X. An ensemble synthesis strategy for fabrication of hollow fiber T-type zeolite membrane modules. *J. Membr. Sci.* **563**, 460-469 (2018).

36. Shin Y*, et al.* Highly Selective Supported Graphene Oxide Membranes for Water-Ethanol Separation. *Sci. Rep.* **9**, 2251 (2019).

37. Yang J*, et al.* Self-Assembly of Thiourea-Crosslinked Graphene Oxide Framework Membranes toward Separation of Small Molecules. *Adv.*

Mat. **30**, 1705775 (2018).

38. Hua D, Rai RK, Zhang Y, Chung T-S. Aldehyde functionalized graphene oxide frameworks as robust membrane materials for pervaporative alcohol dehydration. *Chem. Eng. Sci.* **161**, 341-349 (2017).

39. Hung W-S*, et al.* Cross-Linking with Diamine Monomers To Prepare Composite Graphene Oxide-Framework Membranes with Varying d-Spacing. *Chem. Mat.* **26**, 2983-2990 (2014).

40. Cao K*, et al.* Enhanced water permeation through sodium alginate membranes by incorporating graphene oxides. *J. Membr. Sci.* **469**, 272-283 (2014).

41. Plata-Gryl M, Boczkaj G, Policicchio A, Figoli A, Galiano F, Castro-Muñoz R. Facilitated water transport in composite reduced graphene oxide pervaporation membranes for ethanol upgrading. *Sep. Purif. Technol.* **332**, 125782 (2024).

42. Xiang N, Li L, Wang H, Qian J, Wang TH. GO-enhanced PVA mixed matrix membranes for dehydration of alcohol/water mixture via pervaporation. *J. Mat. Sci.* **58**, 14612-14623 (2023).

43. Castro-Muñoz R*, et al.* Towards the dehydration of ethanol using pervaporation cross-linked poly(vinyl alcohol)/graphene oxide membranes. *J. Membr. Sci.* **582**, 423-434 (2019).

44. Gupta O, Roy S, Rao L, Mitra S. Graphene Oxide-Carbon Nanotube (GO-CNT) Hybrid Mixed Matrix Membrane for Pervaporative Dehydration of Ethanol. *Membranes* **12**, 1227 (2022).

45. Halakoo E, Feng X. Layer-by-layer assembled membranes from graphene oxide and polyethyleneimine for ethanol and isopropanol dehydration. *Chem. Eng. Sci.* **216**, 115488 (2020).

46. Dmitrenko M*, et al.* Pervaporation Membranes Based on Polyelectrolyte Complex of Sodium Alginate/Polyethyleneimine Modified with Graphene Oxide for Ethanol Dehydration. *Polymers* **16**, 1206 (2024).

47. Tseng C, Liu Y-L. Poly(vinyl alcohol)/carbon nanotube (CNT) membranes for pervaporation dehydration: The effect of functionalization agents for CNT on pervaporation performance. *J. Membr. Sci.* **668**, 121185 (2023).

48. Ying Y*, et al.* High-flux graphene oxide membranes intercalated by metal–organic framework with highly selective separation of aqueous organic solution. *ACS Appl. Mater. Interfaces* **9**, 1710-1718 (2017).

49. Zhu T, Xu S, Yu F, Yu X, Wang Y. ZIF-8@GO composites incorporated polydimethylsiloxane membrane with prominent separation performance for ethanol recovery. *J. Membr. Sci.* **598**, 117681 (2020).

50. Cheng X*, et al.* Water-selective permeation in hybrid membrane incorporating multi-functional hollow ZIF-8 nanospheres. *J. Membr. Sci.* **555**, 146-156 (2018).

51. Liu G*, et al.* Pervaporation performance comparison of hybrid membranes filled with two-dimensional ZIF-L nanosheets and zero-dimensional ZIF-8 nanoparticles. *J. Membr. Sci.* **523**, 185-196 (2017).

52. Butt TH, Tamime R, Budd PM, Harrison WJ, Shamair Z, Khan AL. Enhancing the organophilic separations with mixed matrix membranes of PIM-1 and bimetallic Zn/Co-ZIF filler. *Sep. Purif. Technol.* **283**, 120216 (2022).

53. Vinu M*, et al.* Effects of structural crystallinity and defects in microporous Al-MOF filled chitosan mixed matrix membranes for pervaporation of water/ethanol mixtures. *J. Taiwan Inst. Chem. Eng.* **83**, 143-151 (2018).

54. Liu Q, Li Y, Li Q, Liu G, Liu G, Jin W. Mixed-matrix hollow fiber composite membranes comprising of PEBA and MOF for pervaporation separation of ethanol/water mixtures. *Sep. Purif. Technol.* **214**, 2-10 (2019).

55. Xu YM, Japip S, Chung T-S. Mixed matrix membranes with nano-sized functional UiO-66-type MOFs embedded in 6FDA-HAB/DABA polyimide for dehydration of C1-C3 alcohols via pervaporation. *J. Membr. Sci.* **549**, 217-226 (2018).

56. Yang T, Liang Y, Liu G, Wang Z, Tong Y, Li W. Glycine-Modified Co-MOF Pervaporation Membrane to Enhance Water Transporting. *Langmuir* **40**, 12035-12044 (2024).

57. Sorribas S*, et al.* Pervaporation and membrane reactor performance of polyimide based mixed matrix membranes containing MOF HKUST-1. *Chem. Eng. Sci.* **124**, 37-44 (2015).

58. Li H, Zhao C, Ying Y, Zhang W. Improve MOF-801 dispersibility in PVA membranes by a pre-crosslinking strategy for enhanced pervaporation performance. *J. Membr. Sci.* **687**, 122043 (2023).

59. Li Q*, et al.* High efficient water/ethanol separation by a mixed matrix membrane incorporating MOF filler with high water adsorption capacity. *J. Membr. Sci.* **544**, 68-78 (2017).

60. Fu H*, et al.* Mixed matrix membrane for enhanced Ethanol/Water pervaporation separation by incorporation of hydrophilic Zr-MOF NU-

906 in chitosan. *Sep. Purif. Technol.* **318**, 123985 (2023).

61. Song D*, et al.* Incorporating COFs into PDMS matrix for accelerated crosslinking and efficient ethanol recovery. *J. Membr. Sci.* **696**, 122529 (2024).

62. Lin Y-F, Fang Y-X, Xu Z-L, Taymazov D. SUZ-4 zeolite interlayer enhanced thin-film composite pervaporation membrane for ethanol dehydration. *Sep. Purif. Technol.* **314**, 123587 (2023).

63. WANG JT, Huang Z, ZHU YT, Wang SNJTJoC. Poly (vinyl alcohol)/ZSM-5 zeolite mixed matrix membranes for pervaporation dehydration of ethanol and n-propanol. *Chem. Eng. Res. Des.* **47**, 1389-1406 (2023).

64. Wang J-T, Huang Z, Zhu Y-T, Wang S-N. Pervaporative poly(vinyl alcohol)/H-β zeolite mixed matrix membranes for dewatering C2–C3 alcohols. *J. Membr. Sci.* **140**, e54454 (2023).

65. Yang H*, et al.* Functionally graded membranes from nanoporous covalent organic frameworks for highly selective water permeation. *J. Mater. Chem. A* **6**, 583-591 (2018).

66. Wang Y*, et al.* Enhanced ethanol dehydration performance of cationic COFs filled anionic alginate hybrid membranes. *J. Membr. Sci.* **705**, 122906 (2024).

67. Yang H*, et al.* Highly water-selective membranes based on hollow covalent organic frameworks with fast transport pathways. *J. Membr. Sci.* **565**, 331-341 (2018).

68. Wu Y, Ding L, Lu Z, Deng J, Wei Y. Two-dimensional MXene membrane for ethanol dehydration. *J. Membr. Sci.* **590**, 117300 (2019).

69. Xu Z, Liu G, Ye H, Jin W, Cui Z. Two-dimensional MXene incorporated chitosan mixed-matrix membranes for efficient solvent dehydration. *J. Membr. Sci.* **563**, 625-632 (2018).

70. Xu Z, Liu G, Ye H, Jin W, Cui ZJJoms. Two-dimensional MXene incorporated chitosan mixed-matrix membranes for efficient solvent dehydration. *J. Membr. Sci.* **563**, 625-632 (2018).

71. Liu Q*, et al.* Hypergravity field induced self-assembly of 2D MXene in polyvinyl alcohol membrane matrix and its improvement of alcohol/water pervaporation. Chem. Eng. Sci. **140**, e53740 (2023).

72. Wang Z*, et al.* Hydrophilic 2D composite LDHs-MXene pervaporation membrane for highly efficient ethanol dehydration. *J. Membr. Sci.* **703**, 122855 (2024).

73. Li S*, et al.* Highly selective sodium alginate mixed-matrix membrane incorporating multi-layered MXene for ethanol dehydration. *Sep. Purif. Technol.* **235**, 116206 (2020).

74. Song Y*, et al.* Embedding hydrophobic MoS2 nanosheets within hydrophilic sodium alginate membrane for enhanced ethanol dehydration. **185**, 231-242 (2018).

75. Wang J*, et al.* Graphitic carbon nitride nanosheets embedded in poly(vinyl alcohol) nanocomposite membranes for ethanol dehydration via pervaporation. *Sep. Purif. Technol.* **188**, 24-37 (2017).

76. Panahian S, Raisi A, Aroujalian A. Multilayer mixed matrix membranes containing modified-MWCNTs for dehydration of alcohol by pervaporation process. *Desalination* **355**, 45-55 (2015).

77. Cai W, Zhang Q, Yang GQ, Ye H, Song M, Wang Z. High-performance double-separation-layer pervaporation membranes for ethanol dehydration. *Mater. Today Commun.* **40**, 109658 (2024).

78. Lecaros RLG*, et al.* Ionically cross-linked sodium alginate and polyamidoamine dendrimers for ethanol/water separation through pervaporation. *Sep. Purif. Technol.* **275**, 119125 (2021).

79. Sun H*, et al.* Enhanced pervaporation dehydration performance of polyamide thin-film composite membrane with sodium alginate hydrophilic surface modification. *Chem. Eng. Sci.* **140**, e54734 (2023).

80. Cheng F-Y, Zhang X, Lin Y-F, Wu L-K, Xu Z-L, Taymazov D. Mutual-assisted structure of sodium alginate-polyamide membrane for high-efficient dehydration of ethanol. *J Taiwan Inst. Chem. Eng.* **140**, 104564 (2022).

81. Xia Q, Zhu T, Chai Z, Wang Y. TFC membrane with in-situ crosslinked ultrathin chitosan layer for efficient water/ethanol separation enabled by multiple supramolecular interactions. *Adv. Membr.* **3**, 100062 (2023).

82. Zhuang Y*, et al.* Polydimethylsiloxane membrane formed by thiol-ene photopolymerization for bio-alcohols pervaporation. *Sep. Purif. Technol.* **329**, 125138 (2024).

83. Lin Y-F, Ho J-C, Andrew Lin K-Y, Tung K-L, Chung T-W, Lee C-C. A drying-free and one-step process for the preparation of siloxane/CS mixed-matrix membranes with outstanding ethanol dehydration performances. *Sep. Purif. Technol.* **221**, 325-330 (2019).

84. Zhao Z, Zhao G, Tang G, Liu Y, Li P. Designing high-performance pervaporation membranes with hierarchical hydrophobic-hydrophilic coating layers. *Adv. Membr.* **3**, 100073 (2023).

85. Zhang X, Wang M, Ji CH, Xu XR, Ma XH, Xu ZL. Multilayer assembled CS-PSS/ceramic hollow fiber membranes for pervaporation dehydration. *Sep. Purif. Technol.* **203**, 84-92 (2018).

86. Zhang Y, Liu M, Wu Y, Zhao J, Zhou S, Gu P. Zwitterionic polyamide membranes via in-situ interfacial polymerization modification for efficient pervaporation dehydration. *Sep. Purif. Technol.* **333**, 125847 (2024).

87. Ji C-H, Xue S-M, Xu Z-L. Novel Swelling-Resistant Sodium Alginate Membrane Branching Modified by Glycogen for Highly Aqueous Ethanol Solution Pervaporation. *ACS Appl. Mater. Interfaces* **8**, 27243-27253 (2016).

88. Ngamou PHT, Ivanova ME, Guillon O, Meulenberg WA. High-performance carbon molecular sieve membranes for hydrogen purification and pervaporation dehydration of organic solvents. *J. Mater. Chem. A* **7**, 7082-7091 (2019).

89. Mo B*, et al.* Ligand-engineered Zr-MOF membranes with fast water transport channels for alcohol–water separation. AIChE J. **70**, e18368 (2024).

90. Gao G, Zhao Y, Zhu P, Liu H, Guo Y, Zhang X. Self-converted fabrication of a Ni-MOF-74 tubular membrane from nickel-based nanosheets for butanol dehydration by pervaporation. *Cryst. Eng. Comm.* **25**, 6740-6747 (2023).

91. Li Y*, et al.* Rapid synthesis of high-selective Al-rich beta zeolite membrane via an organic template-free route for pervaporation dehydration of water-n-butanol mixtures. *Sep. Purif. Technol.* **308**, 122969 (2023).

92. Wu X*, et al.* Fabrication of hydrophilic and durable Beta zeolite membranes for dehydration of fuel n-butanol by adjusting Al spatial distribution. *Sep. Purif. Technol.* **351**, 128047 (2024).

93. Ueno K*, et al.* Fabrication of pure-silica *BEA-type zeolite membranes on tubular silica supports coated with dilute synthesis gel via steam-assisted conversion. *Sep. Purif. Technol.* **247**, 116934 (2020).

94. Zhang F, Xu L, Hu N, Bu N, Zhou R, Chen X. Preparation of NaY zeolite membranes in fluoride media and their application in dehydration of bio-alcohols. *Sep. Purif. Technol.* **129**, 9-17 (2014).

95. Li Y, Ma N, Liu XF, Zhang BJCG, Design. Synthesis of Dense High-Silica Zeolite Beta Membranes with Controllable Orientation for n-Butanol Recovery from Dilute Aqueous Solution. *Cryst. Growth Des.* ***42*** (2019).

96. Qu F*, et al.* Understanding the effect of zeolite crystal expansion/contraction on separation performance of NaA zeolite membrane: A combined experimental and molecular simulation study. *J. Membr. Sci.* **539**, 14-23 (2017).

97. Wang X, Chen J, Fang M, Wang T, Yu L, Li J. ZIF-7/PDMS mixed matrix membranes for pervaporation recovery of butanol from aqueous solution. *Sep. Purif. Technol.* **163**, 39-47 (2016).

98. Yin H*, et al.* Free-standing ZIF-71/PDMS nanocomposite membranes for the recovery of ethanol and 1-butanol from water through pervaporation. *J. Membr. Sci.* **529**, 286-292 (2017).

99. Unlu DJIPJ. Chitosan/sodium alginate hybrid membranes modified by zeolitic imidazolate framework-90 for pervaporative dehydration of butanol. Iran. Polym. J. **30**, 1239-1249 (2021).

100. Wang S-n, Huang Z, Wang J-t, Ru X-f, Teng L-j. PVA/UiO-66 mixed matrix membranes for n-butanol dehydration via pervaporation and effect of ethanol. *Sep. Purif. Technol.* **313**, 123487 (2023).

101. Xu YM, Chung T-S. High-performance UiO-66/polyimide mixed matrix membranes for ethanol, isopropanol and n-butanol dehydration via pervaporation. *J. Membr. Sci.* **531**, 16-26 (2017).

102. Moriyama N, Nagasawa H, Kanezashi M, Tsuru T. Pervaporation dehydration of aqueous solutions of various types of molecules via organosilica membranes: Effect of membrane pore sizes and molecular sizes. *Sep. Purif. Technol.* **207**, 108-115 (2018).

103. Achari DD, Hegde SN, Pattanashetti NA, Kamble RR, Kariduraganavar MY. Development of zeolite-A incorporated PVA/CS nanofibrous composite membranes using the electrospinning technique for pervaporation dehydration of water/tert-butanol. *New J. Chem.* **45**, 3981-3996 (2021).

104. Dai L*, et al.* Two-dimensional heterogenous channels incorporated by enhanced-surface hydrophilic hollow ZIF-8 nanocrystals for ultrafast water permeation. *J. Membr. Sci.* **661**, 120943 (2022).

105. Zhang Z*, et al.* Vapor-liquid interfacial polymerization of covalent organic framework membranes for efficient alcohol dehydration. *J. Membr. Sci.* **641**, 119905 (2022).

106. Yang H*, et al.* Ultrathin heterostructured covalent organic framework membranes with interfacial molecular sieving capacity for fast water-selective permeation. *J. Mater. Chem. A* **8**, 19328-19336 (2020).

107. Cao C*, et al.* Conferring efficient alcohol dehydration to covalent organic framework membranes via post-synthetic linker exchange. *J.*

Membr. Sci. **630**, 119319 (2021).

108. Yuan J*, et al.* Incorporating covalent organic framework nanosheets via solvent-exchange strategy boosted hybrid membrane dehydration performance. *Sep. Purif. Technol.* **353**, 128315 (2025).

109. Yang H*, et al.* Covalent organic framework membranes through a mixed-dimensional assembly for molecular separations. *Nat. Commu.* **10**, 2101 (2019).

110. Wu G, Li Y, Geng Y, Jia Z. In situ preparation of COF-LZU1 in poly(ether-block-amide) membranes for efficient pervaporation of n-butanol/water mixture. *J. Membr. Sci.* **581**, 1-8 (2019).

111. Liu G*, et al.* High-efficiency water-selective membranes from the solution-diffusion synergy of calcium alginate layer and covalent organic framework (COF) layer. *J. Membr. Sci.* **572**, 557-566 (2019).

112. Dong S, Chen Y, Wang H, Ji Y, Zhao J, Jin W. Synergistically tailoring the hierarchical channel structure of graphene oxide membrane through co-assembly strategy for high-performance butanol dehydration. *J. Membr. Sci.* **678**, 121693 (2023).

113. Song Y*, et al.* Ultrapermeable graphene oxide membranes with tunable interlayer distances via vein-like supramolecular dendrimers. *J. Mater. Chem. A* **7**, 18642-18652 (2019).

114. Liang S*, et al.* Construction of graphene oxide membrane through non-covalent cross-linking by sulfonated cyclodextrin for ultra-permeable butanol dehydration. *J. Membr. Sci.* **621**, 118938 (2021).

115. Zhang Z*, et al.* Engineering fast water-selective pathways in graphene oxide membranes by porous vermiculite for efficient alcohol dehydration. *J. Membr. Sci.* **677**, 121587 (2023).

116. Xiong Z*, et al.* Two-dimensional sub-nanometer confinement channels enabled by functional carbon dots for ultra-permeable alcohol dehydration. *J. Membr. Sci.* **644**, 120069 (2022).

117. Lecaros RLG*, et al.* Homostructured graphene oxide-graphene quantum dots nanocomposite-based membranes with tunable interlayer spacing for the purification of butanol. *Sep. Purif. Technol.* **283**, 120166 (2022).

118. Mao YY*, et al.* Bola-amphiphile-imidazole embedded GO membrane with enhanced solvent dehydration properties. *J. Membr. Sci.* **595**, (2020).

119. Hsieh C-W, Li B-X, Suen S-Y. Alicyclic Polyimide/SiO2 Mixed Matrix Membranes for Water/n-Butanol Pervaporation. *Membranes* **11**, 564

(2021).

120. Lin Y-F, Wu C-Y, Liu T-Y, Lin K-YA, Tung K-L, Chung T-W. Synthesis of mesoporous SiO2 xerogel/chitosan mixed-matrix membranes for butanol dehydration. *J. Ind. Eng. Chem.* **57**, 297-303 (2018).

121. Talluri VP, Tleuova A, Hosseini S, Vopicka O. Selective Separation of 1-Butanol from Aqueous Solution through Pervaporation Using PTSMP-Silica Nano Hybrid Membrane. *Membranes* **10**, 55 (2020).

122. Cong S, Yuan Y, Wang J, Wang Z, Kapteijn F, Liu X. Highly Water-Permeable Metal–Organic Framework MOF-303 Membranes for Desalination. *J. Am. Chem. Soc.* **143**, 20055-20058 (2021).

123. Ji Y-L*, et al.* Roll-to-roll fabrication of large-area metal–organic framework-based membranes for high-performance aqueous separations. *Nat. Water* **2**, 183-192 (2024).

124. Xiao Y, Zhang W, Jiao Y, Xu Y, Lin H. Metal-phenolic network as precursor for fabrication of metal-organic framework (MOF) nanofiltration membrane for efficient desalination. *J. Membr. Sci.* **624**, 119101 (2021).

125. Li Y, Wee LH, Martens JA, Vankelecom IFJJoMS. Interfacial synthesis of ZIF-8 membranes with improved nanofiltration performance. *J. Membr. Sci.* **523**, 561-566 (2017).

126. Zhang M-y*, et al.* Improving the hydrostability of ZIF-8 membrane by biomolecule towards enhanced nanofiltration performance for dye removal. *J. Membr. Sci.* **618**, 118630 (2021).

127. Wang L*, et al.* Layer-by-layer fabrication of high-performance polyamide/ZIF-8 nanocomposite membrane for nanofiltration applications. *ACS Appl. Mater. Interfaces* **7**, 24082-24093 (2015).

128. Karimi A, Khataee A, Safarpour M, Vatanpour VJS, Technology P. Development of mixed matrix ZIF-8/polyvinylidene fluoride membrane with improved performance in solvent resistant nanofiltration. *Sep. Purif. Technol.* **237**, 116358 (2020).

129. Zhao B*, et al.* Polyamide thin film nanocomposite membrane containing polydopamine modified ZIF-8 for nanofiltration. *Colloids Surf. A Physicochem. Eng. Asp.* **612**, 125971 (2021).

130. Basu S, Balakrishnan MJS, Technology P. Polyamide thin film composite membranes containing ZIF-8 for the separation of pharmaceutical compounds from aqueous streams. *Sep. Purif. Technol.* **179**, 118-125 (2017).

131. Yang L, Wang Z, Zhang JJoms. Zeolite imidazolate framework hybrid

nanofiltration (NF) membranes with enhanced permselectivity for dye removal. J. Membr. Sci. **532**, 76-86 (2017).

132. Zhao Y-y, Liu Y-l, Wang X-m, Huang X, Xie YF. Impacts of Metal–Organic Frameworks on Structure and Performance of Polyamide Thin-Film Nanocomposite Membranes. *ACS Appl. Mater. Interfaces* **11**, 13724-13734 (2019).

133. Yuan J*, et al.* Fabrication of ZIF-300 membrane and its application for efficient removal of heavy metal ions from wastewater. *J. Membr. Sci.* **572**, 20-27 (2019).

134. Wang S, Liu J, Pulido B, Li Y, Mahalingam D, Nunes SP. Oriented Zeolitic Imidazolate Framework (ZIF) Nanocrystal Films for Molecular Separation Membranes. *ACS Appl. Nano Mater.* **3**, 3839-3846 (2020).

135. Liu X, Demir NK, Wu Z, Li K. Highly Water-Stable Zirconium Metal–Organic Framework UiO-66 Membranes Supported on Alumina Hollow Fibers for Desalination. *J. Am. Chem. Soc.* **137**, 6999-7002 (2015).

136. Wang X*, et al.* Improving Water-Treatment Performance of Zirconium Metal-Organic Framework Membranes by Postsynthetic Defect Healing. *ACS Appl. Mater. Interfaces* **9**, 37848-37855 (2017).

137. Xu Y, Zhao X, Chang R, Qu H, Xu J, Ma J. Designing heterogeneous MOF-on-MOF membrane with hierarchical pores for effective water treatment. *J. Membr. Sci.* **658**, 120737 (2022).

138. Fang S-Y*, et al.* Construction of highly water-stable metal-organic framework UiO-66 thin-film composite membrane for dyes and antibiotics separation. *Chem. Eng. J.* **385**, 123400 (2020).

139. He Y, Tang YP, Ma D, Chung T-S. UiO-66 incorporated thin-film nanocomposite membranes for efficient selenium and arsenic removal. *J. Membr. Sci.* **541**, 262-270 (2017).

140. Yu Q, Zhou Y, Gao C. UiO-66 regulated thin-film nanocomposite membranes for water treatment. *Desalination* **587**, 117917 (2024).

141. Wang K*, et al.* Development of highly permeable polyelectrolytes (PEs)/UiO-66 nanofiltration membranes for dye removal. *Chem. Eng. Res. Des.* **147**, 222-231 (2019).

142. Zhao P, Li R, Wu W, Wang J, Liu J, Zhang Y. In-situ growth of polyvinylpyrrolidone modified Zr-MOFs thin-film nanocomposite (TFN) for efficient dyes removal. *Compos. B. Eng.* **176**, 107208 (2019).

143. Xiao F, Cao M, Chu R, Hu X, Shi W, Chen Y. Novel Perylene-3, 4, 9, 10-tetracarboxylic dianhydride modified Zr-MOFs/Graphene oxide membrane for dye wastewater treatment. *J. Colloid Interface Sci.* **610**,

671-686 (2022).

144. Ma X-H, Yang Z, Yao Z-K, Xu Z-L, Tang CY. A facile preparation of novel positively charged MOF/chitosan nanofiltration membranes. *J. Membr. Sci.* **525**, 269-276 (2017).

145. Meng YS*, et al.* A high-flux mixed matrix nanofiltration membrane with highly water-dispersible MOF crystallites as filler. *J. Membr. Sci.* **591**, (2019).

146. Meng Y, Shu L, Xie L-H, Zhao M, Liu T, Li J-R. High performance nanofiltration in BUT-8(A)/PDDA mixed matrix membrane fabricated by spin-assisted layer-by-layer assembly. *J. Taiwan Inst. Chem. Eng.* **115**, 331-338 (2020).

147. Li T*, et al.* A novel water-stable two-dimensional zeolitic imidazolate frameworks thin-film composite membrane for enhancements in water permeability and nanofiltration performance. *Chemosphere* **261**, 127717 (2020).

148. Maraddi A, Halakarni M, Manohara Halanur M, Nataraj SK. Fe-MOF induced biopolymer-based sustainable self-cleaning membranes for effective selective separation and wastewater treatment. *SM&T.* **35**, e00537 (2023).

149. Dong W*, et al.* Room temperature aqueous synthesis of defect-engineered MOF-801 membrane towards efficient nanofiltration. *J. Membr. Sci.* **705**, 122946 (2024).

150. Wu M*, et al.* Fabrication of water-stable MOF-808 membrane for efficient salt/dye separation. *J. Membr. Sci.* **686**, 122023 (2023).

151. Fei L*, et al.* Graphene oxide assisted assembly of superhydrophilic MOF-based membrane with 2D/3D hybrid nanochannels for enhanced water purification. *Chem. Eng. J.* **460**, 141694 (2023).

152. Yuan H*, et al.* Large-Area Fabrication of Ultrathin Metal-Organic Framework Membranes. *Adv. Mat.* **35**, 2211859 (2023).

153. Chen YB*, et al.* PVDF/Cu-BTC Composite Membranes for Dye Separation. *Fibers and Polym.* **18**, 1250-1254 (2017).

154. Cheng P*, et al.* Two-dimensional metal−porphyrin framework membranes for efficient molecular sieving. *J. Membr. Sci.* **640**, 119812 (2021).

155. Cai JH*, et al.* Two-dimensional Cu-porphyrin nanosheet membranes for nanofiltration. *Nano Res.* **16**, 6290-6297 (2023).

156. Tan ZK, Gong JL, Fang SY, Li J, Cao WC, Chen ZP. Outstanding anti-

bacterial thin-film composite membrane prepared by incorporating silver-based metal-organic framework (Ag-MOF) for water treatment. *Appl. Surf. Sci.* **590**, (2022).

157. Han L*, et al.* A novel flower-like nickel-metal-organic framework (Ni-MOF) membrane for efficient multi-component pollutants removal by gravity. *Chem. Eng. J.* **470**, 144311 (2023).

158. Bian Q*, et al.* Layered Double Hydroxide-Assisted Fabrication of Prussian Blue Membranes for Precise Molecular Sieving. *Angew Chem Int Ed Engl.* **61**, (2022).
