# Supplementary material for: Facile orientation control of MOF-303 hollow fiber membranes by a dual-source seeding method
Source: Nat Commun. 2024 Nov 26;15:10264. doi: 10.1038/s41467-024-54730-z (PMC11599905; doi:10.1038/s41467-024-54730-z)
Supplement: Supplementary file 3 — Description of Additional Supplementary Files [file 41467_2024_54730_MOESM3_ESM.pdf]

Description of Additional Supplementary Files

File Name: Supplementary Movie 1
Description: Water contact on the MOF-303 membrane.

File Name: Supplementary Movie 2
Description: Ethanol contact on the MOF-303 membrane.
